# Racemization in Post-Translational Modifications Relevance to Protein Aging, Aggregation and Neurodegeneration: Tip of the Iceberg

**DOI:** 10.3390/sym13030455

**Published:** 2021-03-11

**Authors:** Victor V. Dyakin, Thomas M. Wisniewski, Abel Lajtha

**Affiliations:** 1 Virtual Reality Perception Lab (VRPL), The Nathan S. Kline Institute for Psychiatric Research (NKI), Orangeburg, NY 10962, USA; 2 Departments of Neurology, Pathology and Psychiatry, Center for Cognitive Neurology, New York University School of Medicine, New York, NY 10016, USA;; 3 Center for Neurochemistry, The Nathan S. Kline Institute for Psychiatric Research (NKI), Orangeburg, NY 10962, USA;

**Keywords:** D-amino acids, racemization, post translational modification, protein folding, misfolding, aggregation, neurodegeneration, association, cognitive functions, non-equilibrium phase transitions, intrinsically disordered proteins, protein folding energy landscape, brain information processing, adaptive associative learning, cognitive laterality

## Abstract

Homochirality of DNA and prevalent chirality of free and protein-bound amino acids in a living organism represents the challenge for modern biochemistry and neuroscience. The idea of an association between age-related disease, neurodegeneration, and racemization originated from the studies of fossils and cataract disease. Under the pressure of new results, this concept has a broader significance linking protein folding, aggregation, and disfunction to an organism’s cognitive and behavioral functions. The integrity of cognitive function is provided by a delicate balance between the evolutionarily imposed molecular homo-chirality and the epigenetic/developmental impact of spontaneous and enzymatic racemization. The chirality of amino acids is the crucial player in the modulation the structure and function of proteins, lipids, and DNA. The collapse of homochirality by racemization is the result of the conformational phase transition. The racemization of protein-bound amino acids (spontaneous and enzymatic) occurs through thermal activation over the energy barrier or by the tunnel transfer effect under the energy barrier. The phase transition is achieved through the intermediate state, where the chirality of alpha carbon vanished. From a thermodynamic consideration, the system in the homo-chiral (single enantiomeric) state is characterized by a decreased level of entropy. The oscillating protein chirality is suggesting its distinct significance in the neurotransmission and flow of perceptual information, adaptive associative learning, and cognitive laterality. The common pathological hallmarks of neurodegenerative disorders include protein misfolding, aging, and the deposition of protease-resistant protein aggregates. Each of the landmarks is influenced by racemization. The brain region, cell type, and age-dependent racemization critically influence the functions of many intracellular, membrane-bound, and extracellular proteins including amyloid precursor protein (APP), TAU, PrP, Huntingtin, α-synuclein, myelin basic protein (MBP), and collagen. The amyloid cascade hypothesis in Alzheimer’s disease (AD) coexists with the failure of amyloid beta (Aβ) targeting drug therapy. According to our view, racemization should be considered as a critical factor of protein conformation with the potential for inducing order, disorder, misfolding, aggregation, toxicity, and malfunctions.

## 1. Introduction

Under the pressure of previous and emerging results, the idea of the close association between age-related disease, neurodegeneration, and protein racemization shows a meaningful significance [1–7]. The diversity of neurodegenerative diseases possesses both specific and common features [8–485]. In our view, the most common causal mechanism underlying age-related protein misfolding, dysfunction, and aggregation is spontaneous racemization.

The accumulation of misfolded proteins (MPs) is recognized as the most characteristic manifestation of neurodegeneration. The search for the universal mechanism of neurodegenerative diseases eventually considers not only biochemical but also stereochemical processes. Molecular chirality is a critical feature of many biological events in the entire kingdom of life in its normal and pathological forms. Amino acids (AAs) profiles are an effective biomarker (BM) for cardio-genesis [8] and the level of D-amino acids (D-AAs) is increasingly recognized as a novel BM of kidney diseases [9] and neurodegeneration [10]. Chirality is an intrinsic property of peptides and proteins including amyloid beta (Aβ) and microtubule-associated protein TAU, both of which are potent towards the misfolding pathways [11]. It has been shown that dramatic structural perturbations could be triggered by chiral inversions of amino acid chain fragment and any alteration of the physicochemical environment. The effect of chirality perturbations is relevant for the main landmarks of the Alzheimer’s disease (AD): A-β plaques and TAU fibrillary tangles [11]. The racemization of AAs and proteins becomes appreciated as a determinant of most of physiological processes [12–14]. D-AAs have been shown to play an adverse role in the physiology of bacteria ^I^ [15], (I. *D-AAs are found in the cell walls of bacteria, Bacteria are the primary sink for D-AAs contributing to their accumulation in the environments at toxic concentrations* [15] *and insects* [16]).

It is reasonable to expect even more diverse function of D-AAs in animal brain. The biological significance of racemization neuropathogenesis of AD was assessed from as early as year 1994 [17] and remains important [5,18].

### 1.1. Chiral Phase Transitions

The phase transitions associated with stereo-transformation (racemization and isomerization) of peptides and proteins, are driven by the force of increase in the entropy ^II^. (*II. In situations when we can disregard the contribution of other factors such as the enthalpy contribution from the heterochiral interaction* [19].)

The first and second order phase transition ^III^ in AAs, peptides and proteins, despite being theoretically and experimentally explored, are just at the beginning stages of systematic studies [20–23]. (*III. Phase transitions are classified according to Ehrenfest classification* [24]. *The order of a phase transition is defined to be the order of the lowest-order derivative, which changes discontinuously at the phase boundary*).

The concepts of phase transition and chirality transfer are necessary for understanding the biochemistry of AA signaling and protein folding concerning the cell physiology. The effects of chirality transfer from photons to AAs have been recently reviewed [25]. The understanding, description, and interpretation of experiments of protein folding is based on the physics underlying the electron spin system called “spin-glass” paradigm [26–28]. The dynamic behavior of proteins exhibits multiple functional and inactive conformational configurations. The understanding of the structure–function relationship requires the study of kinetic and thermodynamic pathways. At the cellular level, the concept of phase transitions is relevant to the membrane-less organelles (MLO), which represent the coherent structures with the distinct biological functions. Well-known examples include the nucleolus, Cajal bodies, nuclear speckles, cytoplasmic stress granules, P-bodies, and germ granules. MLO molecular structures are environmentally responsive [26,29] and are implicated in the functional protein folding and protein aggregation diseases [30,31]. MLO, peptides, and proteins exhibit various forms of liquid–gel condensations including liquid–liquid and liquid–crystal phase separation, and phase transitions [32,33]. According to [34], the autophagy is considered as a cellular phase transition which maintains the normal cellular functions. The dynamics of the cellular phase transition shown depend on the AAs parameters, however the chirality of AAs is frequently not taken to an account. From the thermodynamic perspective, the existence of a Gibbs potential barrier (energy barrier) between two chiral states is an internal determinant influencing the kinetics of AAs racemization and protein folding. Several external physical and chemical factors influence the rate of stereo-transformation including thermal modulation, photo-stimulation, acoustic-chemical reactions [35], radical reactions, oxidation-reduction sequences, enzyme catalysis, nucleophilic substitutions, and pH of the media ^IV^. (*IV. The rate of aspartic acid racemization in the human connective tissue is about 1% per year* [36–38]).

The widely appreciated “sequence–structure–function” paradigm, postulated by Anfinsen [39], has attracted attention to bio-molecular chirality [23,40]. Three major environmental factors influencing protein conformation are the cytosol, nucleus [30], and cell/organelle membrane. Accordingly, most, if not all, proteins contain segments which have the dual ability to fold into several distinct structures in aqueous and membrane environments [41]. The transport of proteins from the cytosol to the membrane phosphor-lipid or nuclear environment is accompanied by conformational phase transitions. The interplay of the thermodynamic equilibrium and the fundamentally non-equilibrium nature of cellular biochemistry constitutes the basis for the non-equilibrium phase transitions [21,22,33,42].

### 1.2. Biomolecular Chirality

The major classes ^V^ of biomolecules influenced by the phenomena of chirality are: (i) AAs [9,43,44], (ii) peptides, and proteins [45–49], (iii) lipids [50–52], (iv) nucleic acids, (v) DNA, and (vi) RNA [53–55] group. (*V. The most abundant biomolecules belong to four major classes: proteins, lipids, nucleic acids, and carbohydrates. The chirality of carbohydrates (despite its essential role) is beyond the scope of our consideration*).

We will focus mainly on the link between AAs chirality and the protein structure–function relationship. Protein function will be considered with regard to the system of post translational modification (PTM-Sys).

### 1.3. D-Amino Acids in Proteins, Cells, and Neuronal Circuits

The presence of the D-aspartic acid (D-Asp) in myelin and myelin basic protein (MBP) was documented a long time ago [56]. However, the existence of D-AAs in the central nervous system was practically unknown (not discussed) until 2000. Due to the homochirality of biological AAs (L-isoform) in many publications, D-AAs are characterized as “non-biological” [57] or “unnatural” [58]. Presently, diverse D-AAs are found in the body and brain of mammals including, D-serine, D-aspartate, D-alanine, and D-cysteine [59–67]. The current finding suggests that the biosynthetic pathway for D-AAs is conserved from bacteria to mammalian [68]. The aspartic acid and serine are among the most studied AAs due to their distinct role in biochemistry and neuroscience. Both are known as the phospho-acceptors. This fact explains the impact of phosphorylation on the structure of corresponding proteins. AAs racemization (along with deamidation, hydrolysis of peptide bonds, breakage of disulfides, and others) is one of the most active mechanisms in the system of PTM associated with plasticity of protein functions [69,70]. The role of AAs in cell biology, to a significant degree, is determined by interplay between two (L and D) isoforms governed by the spontaneous racemization [18], evolutionarily conserved network of PTM [71–74], and under the environmental factors. Spontaneous, non-enzymatic reactions in proteins are relevant to aging and age-related diseases including AD and cataract. However, the AA-specific mechanisms of spontaneous phase transition are not broadly studied. Recently it was shown that the racemization of the Ser residue occurs preferably in flexible regions of proteins [18]. The translation of peptides/proteins in the eukaryotes utilizes only L-amino acids (L-AAs). The productions of the D-amino acid-containing peptides/proteins through PTM occur via the isomerase enzymes. The isomerization mechanism serves as a yes/no switch of function in the peptide cell- signaling system. The functional significance of racemization is demonstrated by the fact that stereo-transformation modulates the peptide bioactivity in a motor circuit relevant to feeding motor behavior [75].

Growing evidence suggests a vital role of D-AAs not only at the cellular level, but also at the system level; this was shown for immune system [74–78], neuroendocrine system [64,79,80], neurotransmission [81], perception [82], and cognitive functions [83,84]. The ratio of D- AAs to L-AAs increases with the age of the fossil [85] due to the spontaneous racemization ^VI^. (*VI. From a physical point of view, racemization is considered because of phase transition between the R and S enantiomers* [22,86]).

The half-life of the spontaneous and enzymic PTM racemization can range from several days to 100,000 years [87–89].

The racemization has a relevance to the protein/organism aging and age-associated diseases [18], and protein aggregation. Proteins containing D-β- aspartyl (D-Asp) residues were observed in various tissues including cardiac muscle of the heart, blood vessels of the lung, chief cells of the stomach, longitudinal and circular muscle of the stomach, small intestine and large intestine [90]. The presence of free D-Asp in the CNS of rodents and humans was studied [91]. L-Serine (L-Ser) is a major brain metabolite covering functions “from one C-metabolism to transsulfuration, to phospholipid/phosphoprotein function, and to D-serine biosynthesis [92].” The elevated and reduced D-serine level correlates with the progression of many neurological diseases including AD and schizophrenia. D-serine (D-Ser) ^VII^ (non-essential AAs are available from the plant-based diet) is abundant in many regions of CNS including forebrain [93,94] cortex, hippocampus, hypothalamus, amygdala, and cerebellum [95]. (*VII. Serine (Ser) is a non-essential nucleophilic α-AA, encoded by the codons UCU, UCC, UCA, UCG, AGU, and AGC, used in the biosynthesis of proteins* [96,97]).

The distribution of D-Ser and corresponding PLP enzymes suggests an influence on cortico-limbic brain functions [98]. D-Serine (D-Ser) is an endogenous AA implicated in the metabolism of neurons [99], astrocytes [100], oligodendrocytes [101], and microglia cells [102,103] via the variety of signaling pathways. D-Ser and D-Asp were identified as the neurotransmitters. D-Ser is an endogenous co-agonist of the N-methyl-D-aspartate (NMDA) type glutamate receptor at the glycine site [104,105].

Competitive antagonist of AMPA receptor [106] is a key receptor of excitatory neurotransmission in the brain. D-Ser interaction with APP is an essential modulator of the synaptic spine plasticity [107]. In the CNS, D-Ser has a dual (neuronal and glial) origin [105,108]. In addition, D-Ser mediates neurogenesis [109], cellular migration [110], cell proliferation [111], cell death [112], neurotoxicity [113], Neurodegeneration [105], respiratory regulation [114] cardiac activity [115], olfactory perception [116], neuro-endocrine functions [64], immune system [117], learning/memory faculties [118,119] and motor behavior [120]. Proteins and peptides containing D-AAs play an important role in age-related alterations [121–125]. D-glutamic acid (D- Glu) was not believed to be present at any significant level in the brain [84]. However, this was contradicted by the numerous new findings. D-AAs appear to participate in the major biological and neurological mechanisms. D-AAs have been detected in a variety of animal cells’ peptides; these include opiate and antimicrobial peptides from frog skin, neuropeptides from snails, hormones from crustaceans, and venom from spiders. Mammalian hormones and signaling neuropeptides are known as the subject of the functional post-translational racemization (PTM) [126]. However, despite the obvious significance, the role of peptide racemization in cell signaling, aging and neurodegeneration remains the terra-incognito [127]. The presence of D-AAs is detected in brain tissues, cerebrospinal fluid [128], and blood [84,129,130]. Recent measurement suggests that AA levels in brain tissue are typically about 10 to 2000 times higher than in blood [130]. Comparative measurements show that most D-AAs present in the hippocampus are significantly higher in the cortex. Regardless of brain region, the changes in AA chirality cause changes in protein structures (chirality transfer) including forming alpha-helical and beta-sheet structures resulting in changes in metabolic activity and function [19,21,131]. All changes in protein synthesis and degradation are accompanied by sequential spatial (chirality-dependent) transformations. Chirality is also a critical feature of molecular recognition that affects neurotransmission, enzyme activity, and immune functions.

Notably, the processes of protein synthesis and degradation are accompanied by the sequential spatial transformations. D-AAs in organisms are not metabolized by the same pathways as L-AAs and are usually removed by the kidney. In the CNS, an autophagy is known as a pathway for degradation of protein aggregates [132]. It was shown that autophagy, associated with the ubiquitinated aggregates of proteins, was attenuated by a D-Ser in an N-methyl-D-aspartate receptor (NMDAR) pathway [133].

## 2. Chirality at Protein Level: Role in PTMs

### 2.1. Protein Racemization, Aging, Folding, Aggregation, and Degradation

**Figure 1.**
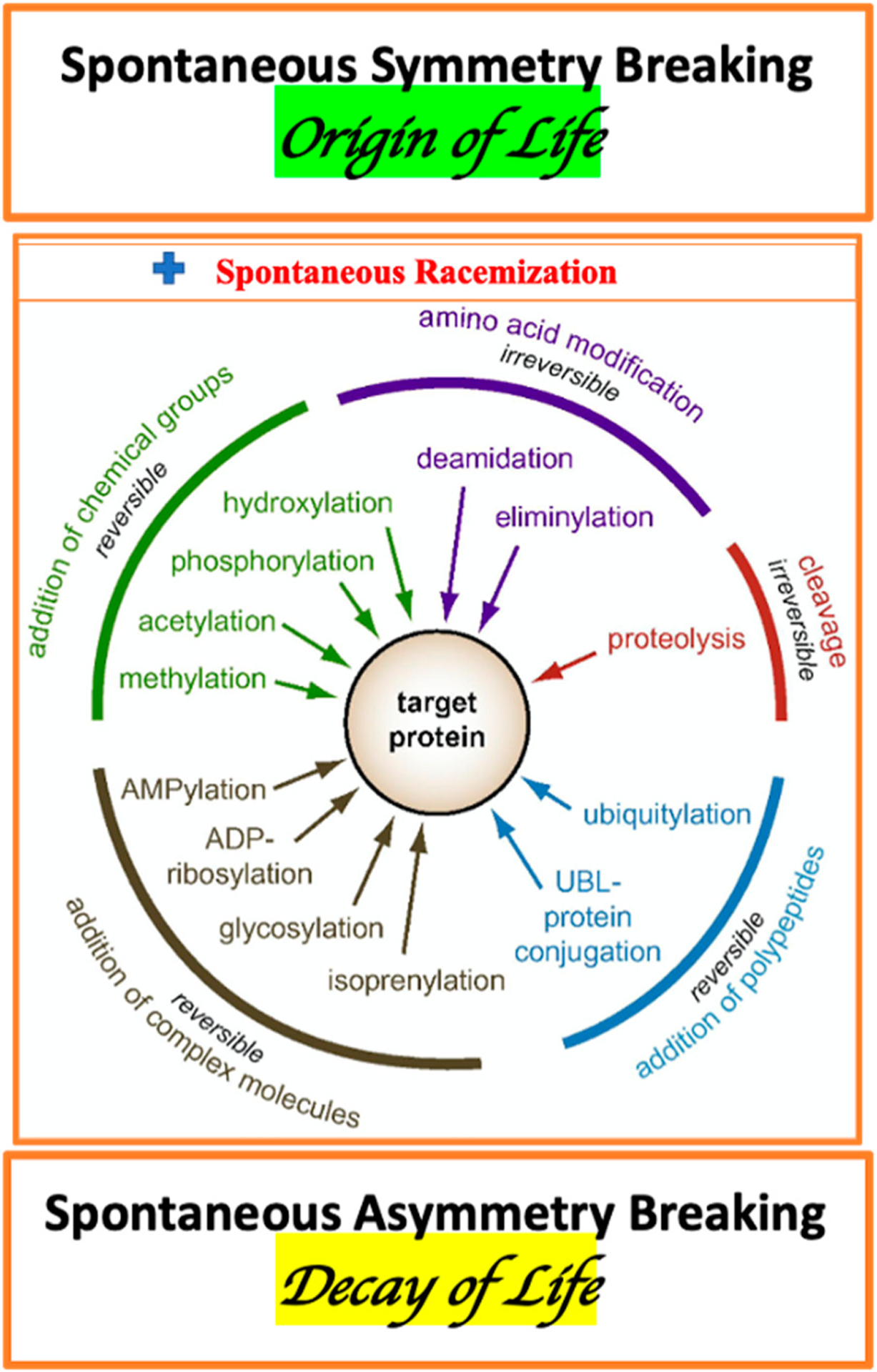
Diversity of post-translational modifications. The spontaneous symmetry breaking in molecular systems resulting in the transfer from the state of thermodynamic equilibrium to the fluctuating non-equilibrium state () is associated with the origin of life. The spontaneous asymmetry breaking in the bio-molecular system resulting in the transfer from the dynamic non-equilibrium state to the state of thermodynamic equilibrium is associated with the decay of life. Part of image is adopted from [134].

The enormous complexity of a living organism, as the essential elements, includes: AAs metabolism, diversity of membrane- and cytosol-associated proteins, variation of proteins stereo-forms, and multiplicity of enzymes of PTM. The traditional view on PTMs should be complemented by the consideration of the spontaneous, irreversible protein conformations associated with AA racemization (Figure 1) [134].

The essential role of AAs is evident beginning from asymmetric cell division [135]. Biological racemization and isomerization are driven by the interplay of spontaneous and enzymatic mechanisms of PTMs. Enzymatic racemization, to a significant degree, is induced by external factors [136]. Three specific and interconnected forms of PTM such as AA racemization (AAR), isomerization (AAI), and phosphorylation (AAP) are routinely used as biomarkers (BM) of peptide degradation and protein aging [137] and aggregation [10]. The bio-catalysts, which decrease the energy barrier for the phase transition, accelerate racemization rate at least by 10^4^–10^5^ times [37]. A relevant example of such catalysts is serine/threonine phosphatase/kinase [138]. Notably, the majority of kinases act on both serine and threonine residues [139]. L-Ser as a central metabolite in cell biology [90] and phosphorylation is a major mechanism of activating/inactivating enzymes, explaining the role of protein kinases in signaling pathways. Due to the above-mentioned facts, we will focus primarily on the role of Ser residue ^VIII^ in the PTM of proteins. (*VIII. The role of D-aspartate (D-Asp) in racemization is covered in a recent review* [67]).

The metabolism of polar AA D-serine (D-Ser) is highly cell-, organ- and brain region-specific [140].

D-Ser metabolism in the brain is regulated by number of enzymes from which we are targeting to enzyme related to racemization and phosphorylation. ^IX^ (*IX. Notably, serine is degraded by hydroxymethyltransferase to glycine. Their role in living organisms is determined by the ability to catalyze a wide range of biochemical reactions including deamination and racemization* [141]).

Pyridoxal phosphate (PLP) enzymes have multiple evolutionary origins [142]. PLP-dependent enzymes exhibit unique catalytic versatility.

#### 2.1.1. Pyridoxal Phosphate Enzyme

PLP enzymes ^X^ are involved in the biosynthesis of protein, glucose and lipid metabolism. We will focus primarily on the Ser racemization. (*X. “The functional specialization of most B (6) enzymes seems to have already occurred in the universal ancestor cell before the divergence of eukaryotes, archaebacteria, and eubacteria 1500 million years ago”* [142]).

The phosphate ion acts as one of the strongest modulators of biomolecular chirality, including Ser-residue. ^XI^ (*XI. Phosphoric acid contains a four-coordinated phosphorus atom. Such molecules are tetrahedral. The four s-bonds with sp3 hybridization of the electron orbitals has tetrahedral orientation*).

The stereo-configurations of Ser residues are sensitive to the effects of metal ions [143], and di-hydrogen phosphate ion {H_2_PO_4_^1-^} [18,144]. Under the influence of the variety molecular environments, Ser undergos racemization as internally bound residues of peptides and proteins, providing an opportunity for the normal and pathological protein degradation and for appearance of D-enantiomers in mammalian cells [145]. However, the research devoted to neurodegenerative diseases has not studied the involvement of Ser racemization (along with the other forms of PTM) in the pathological protein misfolding and aggregation. In this review, we illustrate how current studies have examined racemization. The five families of PLP enzymes include: type I—aspartate aminotransferase family, type II—tryptophan synthase family, type III—alanine racemase family (TIM-barrel), type IV—D-amino acid aminotransferase family, type V—glycogen phosphorylase family [146]. The functions of PLP include influence on pi-electron systems and the chemical properties of contiguous sigma bonds [147]. The PLP acts as a coenzyme in all transamination reactions, and in certain decarboxylation, deamination, and racemization reactions of AAs. The aldehyde group of PLP forms a Schiff-base linkage (internal aldimine) with the ε-amino group of a specific lysine group of the aminotransferase enzyme. Transamination is involved in the ketamine production [148]. PLP is also involved in various beta-elimination reactions such as the reactions carried out by serine dehydratase [149]. Among the functions relevant to PLP activity are the following: (a) to react with glutamate, which transfers its alpha-amino group to PLP to make pyridoxamine phosphate (PMP) and (b) to provide the catalytic functions for PLP enzymes including serine racemase (SerR).

#### 2.1.2. Serine Racemase

The attention to the significance of racemization in neurodegenerative diseases [17] and its association with proteins’ aggregation emerged a long time ago. Due to the chain of essential facts, we have mainly concentrated on the racemization ^XII^ of Ser residues in proteins involved in the neurodegeneration. (*XII. The association of serine/threonine phosphorylation with protein disorder is a common landmark of neurodegeneration* [150–154]).

Among them are: APP(Aβ) [155,156], TAU [157–159], α-Synuclein (α-Syn) [160,161], and prion protein (PrP) [160,162] containing Ser residue in the AAs monomer sequence. An increasing number of experimental findings proves an assumption of the pivotal role of serine racemase (SerR) in the neuronal activity and neurodegeneration. Racemization of protein-bound AAs (including Ser) is important in the aging and pathologies of proteins. Ser undergoes racemization as internally bound residues of functional proteins [163]. Racemization of Ser and Asp residues differently impacts the hydrolysis of proteins. Serine racemase (SerR) is the brain-enriched glial (astro-and micro-glia) cells PLP enzyme [145,164–166] which catalyzes racemization of L-Ser to D-Ser. The catalytic mechanism of the SerR is similar to the alanine racemase. The unprotonated PLP-substrate intermediate is stabilized by the interaction of active-site residues with water molecules, contributing to the enzyme’s electrostatic environment. SerR is a homodimeric pyrixidal 5′-phosphate (PLP) dependent enzyme catalyzing beta-elimination of both L- and D-serine to pyruvate and ammonia [167]. The homo-dimer of SerR (each monomer 340 amino acids) consists of two domains (a small and a large) connected by a flexible loop [168,169]. Both mouse and human SR contains functionally active Ser residue (ValSerCys sequence) at their C-terminus [92,169–171]. This fact suggests that SR activity itself can be modulated by non-enzymic L-serine racemization. The ValSerCys sequence resembles the (PDZ) domains for binding to PSD95 [170]. SerR is activated by binding to the PDZ6 domain of Grip. This complex of molecular interactions represents the pathway for modulation of synaptic spine activity through PSD95. Full activation of SeR requires binding to the remaining part of the C-terminal region of GRIP [170]. The combination of above-mentioned facts provides the idea of the multiple pathways connecting Ser racemization with synaptic spine function through PSD95 [172,173]. It is notable that substrate of the SerR-protein PSD95 contains multiple Ser residues as active sites of PTM. Two evolutionarily conserved sites of serine phosphorylation (Ser-415 and Ser-418) signify the sensitivity of PDS95 signaling system to Ser racemization. [174]. The association of D-Ser and SeR with PSD-95 maintain an overall stability of glutamatergic synapse [175,176]. Ser-R regulated by many cofactors including phosphorylation [98]. Experimental results indicate that PKC phosphorylates SerR in serine residues and regulates D-Ser availability in the brain [177]. In more general terms, the inherent interaction between racemization and phosphorylation, in our view, is relevant for the regulation of physiological and pathological mechanisms of protein folding. ^XIII^ (*XIII. The role of glycosylation in protein folding has been considered in a literature review* [178]).

This hypothesis is supported by the fact that A-Beta aggregations into filaments become irreversible due to the combined force of several PTMs. The interplay between racemization and phosphorylation promotes incorporated A-β dimers and tetramers into resistant to proteolytic degradation filaments [179].

PLP enzyme serine racemase (SerR) catalyzed D-Ser synthesis [180,181] and D-amino acid oxidase (D-AAO) catalyzed D-Ser degradation [182]. SerR, in addition, degrades L- and D-Ser to pyruvate and ammonia. As a residue prone to racemization and phosphorylation Ser is a primary suspect in protein aggregation. The activation barriers of Ser racemization, estimated in the presence of dihydrogen phosphate ion (H^2^PO^4-^), found were consistent with spontaneous rate of reactions occurring at physiological temperature [36,183]. The AAs racemization is driven by spontaneous (non-enzymic) and enzymic process. The rates of AAs racemization in proteins are temperature/protein-dependent, and usually slow under physiological conditions. The progress in the measuring of AAs racemization rate elaborates the concept of protein aging. For aspartic acid (Asp) the rate of racemization occurs over the range from several days to more than 15,000 years [18,38,85,88,184–186]. The L-D conversion of aspartic acid in the proteins of human dental enamel (such as dentine) is relatively fast (about 8% conversion in 60 years) and correlates with a chronological age of the organism [187]. The Asp racemization was seen during ageing and cataract formation [188]. Due to a well-known succinimide-mediated mechanism, the Asp residues are the most racemization-prone [189–192]. Ser is known as one of the main AAs involved in racemization [145,193]. Accordingly, within the lifetime, these AAs residues of long-lived proteins (LLPs) are progressively racemized [18]. In the age-related diseases, this racemization process can be related to protein misfolding and dysfunction [194–196]. The idea of conjugality of SerR activity and APP-related AD pathology is supported by the fact that the level of a brain serine racemase expression can be induced by several seine-containing peptides including the APP fragments such as sAPP [197] and pro-inflammatory stimulus including Aβ peptide [198]) and AP1 [197–199]. SerR is a component of the complex network PTM. The Ser residues of SerR are the targets of several protein kinases including PKC and PICK1 [200].

#### 2.1.3. Serine Protease

Most of the metabolic enzymes recognize only substrates (proteins and peptides) composed exclusively of L-AAs [10]. Serine proteases (SerPs) are ubiquitous in all organisms. Insights into the atomic level of SerPs structure–function link reveal the significance of the catalytic Ser motions [201,202]. Notably, an evolutionarily conserved catalytic domain Ser–His–Asp contains Ser residue [203,204] providing sensitivity to the AAs racemization. It is in the agreement with a well-known fact that incorporation D-AAs into peptide chain diminishes their susceptibility to proteases [205]. The combination of experimental facts, as mentioned earlier, emphasizes a critical role of racemization on enzyme–substrate interaction. The functions of SerPs are closely associated with the degradative pathway of many PLP enzymes [206,207]. SerPs, representing about one-third of all proteases, serve as essential component of the intracellular and extracellular catalyzing hydrolytic reactions [208]. SerPs participate in many physiological processes including food digestion, embryo development and immune defense [209]. The fact that SerR is degraded through the ubiquitin-proteasomal system [210] and regulated by phosphorylation [180] points to crass-talk between Ser-associated forms of PTM emphasizing the physiologic importance of Ser residues. Quantum calculations reveal the mechanism of SerPs action including four specific residues in a water-containing environment [211–213]. SerPs enzymes are involved in the proteolysis of the diverse group of signaling peptides and functional proteins [204] including Aβ [214], APP ^XIV^ (*XIV. Recent studies have reported that many proteases, besides the canonical α-, β- and γ-secretases, cleave the APP* [214], *Aβ peptides* [215], *TAU, and tubulin* [216–218]).

In humans SerPs comprise several groups: plasmin, acylpeptide, hydrolase, and myelin basic protein (MBP) [215,219]. The group of rhomboid SerPs belongs to the family of intramembranous proteases ^XV^ (*XV. BACE1 is, known as membrane-associated aspartic protease 2* [220] and plays a key role in major cellular processes [221–223]).

SerPs are involved with the degradation of aberrantly folded proteins [222,224]. Notably, the sequential cleavages of APP occur by β- and γ-secretases. Both secretases are members of a new class of intramembrane-cleaving proteases (I-CliPs). These proteases include β-secretase 1 (BACE1) the Rhomboid family of SerPs, and two aspartyl proteases: the signal peptide peptidase (SPP) and γ-secretase. “In sharp contrast to Rhomboid and SPP that function as a single component, γ-secretase is a multi-component protease with complex assembly, maturation and activation processes” [221]. Aβ peptides are subject to proteolytic degradation by a family of peptidases and proteinases known under the common name Aβ-degrading proteases (AβDP) [215]. Among them are SerPs, which are ubiquitous in all organisms. As most proteases SerPs are chiral, meaning they distinguish between L- and D-enantiomers of the substrate. Apparently (based on the summary of experimental facts) that SerPs activity and protein folding and aggregation can be strongly affected by the Ser racemization.

#### 2.1.4. D-Amino Acid Oxidase

The metabolism of D-AAs in a healthy organism is modulated by two stereo-specific enzymes: D-amino acid racemase (in the synthesis), and D-amino acid oxidase (D-AAO) ^XVI^ in degradation. (*XVI. D-AAO regulates NMDA receptor function through AAs*).

As a detoxification enzyme, the D-AAOs (in the presence of molecular oxygen) selectively degrade (by oxidative deamination) only D-enantiomers [28]. DAAO is involved in many aspects of cell physiology. As a detoxification enzyme, the D-AAO (in the presence of molecular oxygen) selectively degrades (by oxidative deamination) only D-enantiomers. D-AAO involved in many aspects of cell physiology [225]. As a D-AAs degrading enzyme, D-AAO is associated with many disease conditions including amyotrophic lateral sclerosis [226] and schizophrenia [227]. In the human brain, DAO expression was found to be both age- and brain region-dependent [140,228].

### 2.2. Protein Aggregation and Neurodegeneration

The current review focuses on the most common and basic mechanism of protein aggregation-molecular chirality and racemization. Protein aggregation is a prominent feature of many protein misfolding diseases causing neurodegeneration. Among them are Alzheimer’s (AD), Parkinson’s (PD), Huntington’s (HD) diseases, amyotrophic lateral sclerosis (ALS), Lewy Body Dementia (LBD), progressive supranuclear palsy (PSP), spongiform encephalopathies (SE), cataracts, musculo-skeletal disease (MSD) and demyelination diseases (DD) [229–235]. In our view, racemization should be considered as a common and critical factor of protein conformational stability, potency to aggregation and toxicity [105]. In this review, we focus predominantly on AAs racemization. The first observation of AAs racemization was reported a century ago. The review of the earlier works can be found in [236,237]. AAs undergo spontaneous and catalytic racemization. Two distinct forms of catalytic racemization are base- and acid-catalyzed [19]. In the 1970s–1980s, racemization was used to determine the age of AAs in the biological systems [238–241]. However, the association of AAs racemization with the pathological protein aggregation and the neurodegeneration, during this period, did not attract attention. Structurally ordered protein aggregates (amyloids) are found in all living organisms including the bacteria [7], plants [242] and animals [243]. Contrary to the common view, in humans they are involved not only in the aggregation-related diseases but also in normal physiological activities associated with cognitive function.

#### 2.2.1. Structurally Ordered Proteins

The comparative studies of amyloids structures (fibrillar, cross beta-sheet quaternary forms) in the bacteria, fungi, insects, invertebrates, and humans reveal two sub-sets of fibrils: pathological and functional [244,245]. New findings suggest that the current knowledge regarding the variety of structural conformations of Aβ is far from complete and probably not enough for the development of an efficient therapeutic strategy. The existence of micelles in the fibrillo-genesis of beta-amyloid peptide was proved by experimental results [246–248]. From the bio-physical point of view, it is the spatial distribution of positively and negatively charged domains over surface of protein and spatial orientation of electron spin that tunes the aggregation behavior of proteins [249,250]. Among the broadly studied protein aggregations are inclusion bodies, amyloid fibrils, and other misfolding aggregates. Most protein aggregates contain the secondary structural components such as helical and β sheets elements [251]. The primary hypothesis assumes that aggregation involves the partially folded intermediates and specific intermolecular interactions (molecular chaperones).

The discovery of D-Ser in the chaperone proteins (αA-crystalline) suggests an essential role of the molecular environment in the mechanism of protein folding and interaction [188,252].

#### 2.2.2. Intrinsically Disordered Proteins

The elegant results of such attention to the nature of non-equilibrium phase transitions in proteins is the concept of intrinsically disordered proteins [234]. Intrinsically disordered (ID), intrinsically unstructured protein (IU), or natively unfolded (NA) protein or domain lack a unique three-dimensional structure and exist in a variety of conformations that are in dynamic equilibrium under physiological conditions. It was recognized that some functions of proteins can be associated with the dynamically unstructured conditions. About 40% of eukaryotic proteins have at least one long (>50 AAs) disordered domain [253–255]. The mutations within intrinsically disordered regions (IDRs) increase the aggregation propensity, such as those seen in the amyloid β-peptide, α-synuclein, huntingtin, prion protein, and TAU have been directly linked to variety of above previously mentioned IDs. TAU is most studied IDP [256] but unfortunately the experimental design and analysis of experimental results frequently do not involve consideration of D-AAs residues. In this situation, the classification of IDP based on the AAs sequences reveals the roles of L- and D-Ser in the structure and functions relationships [257,258]. Recently the theoretical framework was introduced for the use of D-amino acids as a universal tool to the exploration the aggregation pathways of IDPs [11]. The study of non-equilibrium phase transition [22] in IDP and the role of L- D- AAs substitution is a matter of urgency. The rate of racemization of amino acids (AAs) is temperature dependent and under influence of external physical fields can be altered in the order of 10^4^–10^5^. The balance between physiological protein folding and aggregation relies on the competition between two pathways. The factors promoting aggregation prevent natural folding and vice versa [251]. The chirality of amyloid fibrils is well established [259,260]. The earlier intuitive ideas on the link between the spontaneous phase transition (chirality transfer and chirality inversion) between the polymorphic forms of the amyloid fibrils ^XVII^ and protein aggregates have gradually gained objective confirmation [18,260–263] (*XVII. The supramolecular chirality of the amyloid fibrils can be registered by variety of the methods including the microscopy (electron (EM), transmission (TEM), and scanning electron (SEM) microscopy) and vibrational circular dichroism (VCD)* [261–264]).

#### 2.2.3. Racemization Role in Protein Folding, Aggregation and Neurodegeneration

Several authors suggested that the “presence of the isomers may be one of the triggers of abnormal aggregation and may induce the partial unfolding of protein leading to a disease state” [88,90]. Recently, it was shown that amyloid fibrils of different nematic phases, including chiral protein-based systems, undergo liquid-crystalline phase transitions [265]. Spontaneous and enzymatic racemization reactions influence protein misfolding and aggregation associated with aging and age-related diseases [142,180]. The exploration of the AAs racemization [28,266] and protein aggregation phenomena within the bacteria cells opens an evolutionary perspective [267].

The amyloid-like properties of inclusion bodies and protein aggregation in bacterial cells have become the point of attention [268,269]. Spontaneous and enzymatic racemization reactions have relevance to the protein misfolding, aggregation associated with aging, and age-related diseases [267]. Gene mutation and spontaneous racemization of Aβ, TAU, PrP, Huntingtin, and alpha-synuclein proteins are determined as major limiting factors in natural peptide synthesis and incorporation of the peptide into functional proteins, leading to abnormal phosphorylation, aggregation, and deposition [14,270–273]. The investigation of the biochemistry of mandelic acid-base molecular structures reveals thhe effect of relative chirality of monomers on the aggregation patterns. The structure of dimers and supramolecular aggregates is strongly affected by the relative monomer chirality [274–276]. At the molecular orbitals level, the transmission of chirality occurs through the cooperation of hydrogen bonding and π − π stacking interactions [271,275]. The recent studies of L- and D- (Aβ) 42 peptide enantiomers confirm an assumption that the chirality of AAs is the key determinant of the oligomer’s solubility and aggregation [277,278].

## 3. Hypothesis of Protein Aggregation and Neurodegeneration

The metabolism of Aβ peptides (full length and the truncated forms), despite being a major target of neurodegenerative studies, remains to be elucidated. The various PTMs of Aβ peptides were explored and discussed during recentdecades, including racemization/isomerization, oxidation, nitration, truncated reaction, glycation, and glycosylation. However, “no common perception of the essential foundation of the AD pathology was determinate” [131]. The overall structure of Aβ−40 and Aβ−42 peptides contains the distinct domains characterized by specific physicochemical properties and 3D structures [279]. In particular, Aβ−42 peptide contains hydrophobic regions (such as KLVFF residues 16–20) [280] and α-helical (right-handed) domains (13–26 residues) [281]. The intuitively attractive therapeutic strategy against amyloid-beta aggregation is based on assumption that drugs should exhibit molecular chirality [280]. In other words, the folding pathway of Aβ−42 is valued for its sensitivity to the chirality of the immediate molecular environment. The chirality of molecular environment, in turn, is mediated by the mechanism of PTM. Therefore, the concept of AAs racemization allows the confluence of several hypotheses of protein aggregation and neurodegeneration.

### 3.1. Amyloid Cascade Hypothesis

The amyloid cascade hypothesis links the misfolding of the Aβ peptide to the cause of AD [282]. The spatial conformations of Aβ peptides are peptide-length specific [283,284]. The aggregation is contributed by multiple pathways directly related to the stereochemistry of the Aβ peptide or indirectly through interaction with aggregated TAU protein [284–286] and membrane lipids [287,288]. The Aβ peptide is prone to aggregation through calcium dysregulation [289], oxidative stress [290], phosphorylation [152,291,292], and inflammation [293]. The amyloid cascade hypothesis emphasizes the role of amyloid- β (A-β) peptide aggregation in the pathogenesis of AD [294].

### 3.2. Glutamate Toxicity Hypothesis

The excitotoxicity of extracellular glutamate was associated with the numerous neurological diseases including ALS, AD, PD, HD, LBD, PSP, and cataracts. We will return to this hypothesis at the consideration of link between D-Ser and functions of glutamate receptors including NMDAR [295], mGluR [296], and AMPAR [106].

### 3.3. Post Translational Modification Hypothesis

The various PTMs of Aβ peptides were explored and discussed, including oxidation, nitration, truncated reaction, glycation, and glycosylation [130,297]. The Aβ is the product of the normal proteolytic processing of AβPP, a type 1 trans membrane glycoprotein [298] whose gene is located on chromosome 21 [299,300]. The class of intrinsically disordered (ID) amyloid peptides and proteins includes Aβ, TAU, islet amyloid polypeptide, and α-Synuclein [301–305]. Amyloid consists of linear, unbranched protein or peptide fibrils of approximately 100 Å diameters.

The fibrils are composed of a wide variety of proteins that have no sequence homology and no similarity in three-dimensional structures. However, fibrils share a common secondary structure, the beta-sheet [302–306]. PTM in general and in racemization increases the heterogeneity of protein conformation and, consequently, the diversity of a protein’s physiological functions and pathological pathways. The illustration of protein chirality-related effects is the physiological stereo transformations of the amyloid precursor protein (APP) and TAU. The processing of APP and PTM of amyloid-beta (Aβ) peptides along with the oxidation, phosphorylation, nitration, pyroglutamylation, and glycosylation include racemization and isomerization [307,308]. The racemization of Aβ peptides generates APP fragments with different physiological and pathological properties modulating disease progression. It is important to emphasize the interconnected chain of events.

- The accumulation of Aβ and TAU trigger the perturbations in the glutamatergic synapse.
- The pre-synaptic and post-synaptic sides of the glutamatergic synapse are modulated by many D-AAs including D-Ser [104,309,310]
- The regional distribution of D-Ser in the brain follows the distribution of NMDA receptors [311,322].
- D-Ser is found in the synaptosomal fraction isolated from rat brain tissues [104,313].
- Glial-neuronal interaction ^XVIII^ at the glutamatergic synapses is a major influence of short- and long-term potentiation responsible for different memory functions [314,315] (*XVIII. S-NMDAR receptors primarily use D-serine, released by neighboring astrocytes* [314]).
- SerR is transcriptionally induced by sAPP [197].
- The NMDA receptor hypofunction is associated with aging, neurodegeneration leading to the impairments of memory, learning and psychosis [316].
- Modified form of TAU in PHFs contains more D-Asp that TAU proteins from normal adult brains (N-TAU) [317]. The chain of the physiological molecular events is inherently linked to the mechanism of racemization. The concept of AAs racemization allows the confluence of three above-mentioned hypotheses of neurodegeneration and protein aggregation. In our view, the racemization is the common relevant factor for the widely circulating hypotheses including the amyloid cascade hypothesis, glutamate toxicity hypothesis [229,231] and hypothesis associated with the functions of PTM network. In support of universal significance of AAs chirality and racemization is speaking the facts that a gradual racemization of peptide and proteins has been observed in aging populations [318], and that mixed chirality proteins evade the known pathway of proteosomal degradation [319]. Notably, the age-related racemization of AAs is critical for function of both the enzymes and their substrates.

## 4. Racemization Role at Molecular, Cellular, and System (Organ) Levels

### 4.1. Molecular Level

#### Aberrant PTM Resulting in Resistance to Proteolytic Degradation

The mechanisms of protein modifications comprise co-translational, post-translational and spontaneous types. The stereo selectivity of the translational machinery of protein synthesis provides reliable defense against the accidental incorporation of D-AAs. It was shown that chirality discrimination occurs at three successive steps (initiation, elongation, and termination) involving tRNA and ribosomal interaction [320–322].

The homochirality sustained by the translational machinery provides the platform for the activity of the post-translational modification (PTM).

Following the translation of polypeptide chain, ^XIX^ determined by the DNA, most proteins undergo evolutionarily conserved PTM (*XIX. Even changing just one AA in a protein’s sequence can affect the protein’s overall structure and function*).

For example, phosphoserine is a component of many proteins as the result of post translational modifications [323,324]. Neuronal protein phosphatases in cell signaling pathways are represented by phosphoserine phosphatases ^XX^ (PSPs) [325]. (*XX. Full activation of SerR requires binding to the remaining part of the C-terminal region of GRIP* [170]).

There are several mechanisms of PTM including: covalent modifications (phosphorylation [326,327], methylation [328], glycosylation ^XXI^ [329,330]), proteolysis [331], oxidation [332–334], deamination [335], cross-linking [336,337], and racemization (enzymic and non-enzymic). The phosphorylation of D-AAs residues is a common way to regulate the activity of proteins. (*XXI. “Glycosylation is one of the most common, and the most complex, forms of post-translational modification of proteins”* [337]).

The source of phosphate for phosphorylation is ATP. The cross-linking PTM is observed for APP and TAU proteins [337]. The variety of forms of PTM is considered as the mechanism of adaptation to the stereochemistry of the environment. For the purpose of our review, it is essential to note that PTM was linked to abnormal deposition of peptides in the brain tissue [338]. The structural heterogeneity of peptide in the aggregations was associated with structural rearrangements of the L- and D- isoforms of aspartyl residues. The localization of D-AAs in peptide chain (N- and C-terminus, or intermediate position) provides an opportunity for modulation of diverse pathways of PTM. All known mechanisms of PTM directly or indirectly involve racemization. D-AAs containing peptides (characterized by altered 3-D shape and charge distribution) show an increase in resistance to proteolytic degradation of molecular aggregation comprised of insoluble depositions [331,339,340] The stereochemical nature of PTM is most evident in the case of racemization. The modulation of AAs chirality influences the spatial transformation of proteins and distribution of hydrophobic/hydrophilic domains. The increase in “hydrophobicity” results in deposition from aqueous media [341]. This in turn changes the balance of soluble and insoluble components in the cytoplasm and in the intracellular space. The physiology of phosphorylation, oxidation, glycation, and ubiquitination is inevitably influenced by the age-associated, cell-specific racemization [36,100,342–346]. Thus, racemization of AAs could be a common mechanism for many pathogenic pathways. Protein misfolding and aggregation are responsible for the brain neurofibrillary tangles (NFT) and neuritic plaques (NP). Protein aggregates and excitotoxicity, representing the common landmarks of major NDs including ALS, AD, PD, LBD, PSP, HD and cataracts, are inevitably linked to the AAs racemization. For the heat shock proteins in the lens (αA-crystallin (αA) and αB-crystallin (αB)) it was shown that an aggregation and deposition is significantly contributed by several types of PTM. Among them are oxidation, C- and N-terminal truncation, deamidation, phosphorylation, and methylation [333,334]. As we mentioned before, many forms of PTM are directly associated with the protein racemization [194].

### 4.2. Cellular Level

#### 4.2.1. D-Seine and NMDA-Dependent Neurotransmission

NMDA receptor and corresponding neurotransmitters are one of the best examples of stereoselective interaction. D-AAs (including D-Ser and D-Asp) are involved in many aspects of the brain’s excitatory and inhibitory neurotransmission [67]. For example, in neurons D-Asp serves as a neurotransmitter delivered to NMDA receptor site in synaptic vesicles [67,347,348]. The convincing way to illustrate the role of AAS chirality in the glutamatergic system is to review NMDA receptor ligands.

NMDA receptor agonists and partial agonists include: L-glutamate, D-glutamate, N-methyl-D-aspartate (NMDA), N-methyl-L-aspartate, D-aspartate, L-aspartate, and many others [60,66,67] The co-agonists of NMDA receptors include D-serine, L-serine, D-alanine, and L-alanine [62,63]. In the central excitatory and inhibitory synapses of the mammalian brain, L- and D-isoforms of Ser play a key role in signal transduction [193]. D-Ser participates in the synaptogenesis, synaptic transmission (NMDA and AMPA [106]), synaptic remodeling [349], and spine plasticity [107,350]. In the tripartite synapse, the downregulation of neuronal D-Ser levels under any pathological conditions is naturally associated with an enhanced production and release of D-Ser by astrocytes. The physiology of neuronal-astroglia loop is regulated by the interplay of the enzymes including SerR and D-AAO. The disruption of the natural feedback mechanisms regulating cell–cell and enzyme–enzyme interaction can accelerate the neurodegeneration [351]. As an example, we can point on moto-neuronal death in the mouse model of amyotrophic lateral sclerosis [352]. Astrocytes (as the source of D-Ser) possess the vesicles sequestering and storing D-Ser as gliotransmitter [353,354] The Ser-containing vesicles undergo calcium-dependent exocytosis modulating synaptic NMDA transmission. The activation of opening of the NMDA receptor requires coincidence in occupation of the glutamate and the glycine site. At the post synaptic dendritic spines, an NMDA-dependent endocytosis of GABAB receptors requires the phosphorylation of its intracellular C terminus domain serine 867 residue (Ser867) in the intracellular C terminus [355]. The attenuation of the neuronal nicotinic acetylcholine receptors (nAChRs) by receptor antagonist alters the function and expression of SerR [356] suggesting involvement of cholinergic circuits in modulation of D-serine level. The review of current publications related to the mechanism of Glu-receptors internalization suggests an active role of D-Ser in mediating NMDA and AMPA receptors endocytosis [357]. Pathological roles of free extracellular D-Ser mediating NMDA receptor overactivation are suggested in studies using in vitro culture systems [358]. The internalization of cell membrane receptors involving Ser activity is observed in many cell types. We provide several examples. (1) In liver, parenchymaglucagon-mediated internalization of the serine-phosphorylated glucagon receptor is mediated by serine-phosphorylated residue [359]. (2) Phosphorylation Ser-789 in the C-terminal tail of fibroblast growth factor receptor 1(FGFR1) is required for receptor endocytosis [360]. (3) The low-density lipoprotein receptor-related protein (LRP), which participates in endocytosis, signaling pathways, and phagocytosis of necrotic cells, is mediated by phosphorylation of the serine residues within the LRP receptors cytoplasmic domain by PKCα [361]. (4) The carboxyl-terminal Ser residues (Ser-355, Ser-356, and Ser-364) play a critical role in G protein-coupled receptor kinase (GRK)-mediated phosphorylation and desensitization of β_2_-adrenergic receptors (β_2_Ars) [362]. (5) The endocytosis of CD4 (cluster of differentiation antigen is activated by Ser phosphorylation [363,364].

#### 4.2.2. Racemization-Prone Ser Residues

The Ser residues appear more racemization-prone than other residues [5]. The racemization–deracemization dynamics are a natural discriminant between the healthy physiological state, aging, and disease condition [18,267]. For example, D-Ser promotes adult hippocampal neurogenesis enhancing cell proliferation and increase in the survival of new neurons [109]. At the same time, D-Ser is known as a key determinant of glutamate toxicity [352] and D-AAO enzyme-mediated metabolism, results in reduction in the reactive oxygen species (ROS) [365].

SerR/D-Ser/NMDA-receptor pathway is recognized as a regulator of apoptosis and necrosis shift during different forms of excitotoxicity involving microglia activation [102,366,367]. SerR belongs to the class of co-factor-dependent AA racemase enzymes. Accordingly, the activity of SerR is mediated by many co-factors including divalent cations (Mg. Mn, Ca, Fe Ni Cu, Co and Zn) [368,369], nucleotides (ATP, ADP or GTP) [180], and sulfhydryl groups [369,370].

The binding of ATP to serine racemase links the production of D-serine to the energy metabolism [157,371–373]. Consequently, racemization, of any origin (spontaneous and induced), will interfere with the cell aerobic metabolism [374]. The presence of D-AAs detected in plants, bacteria, and mammals is associated with the diverse range of biological functions [375]. “The levels of D-Ser in the brain are higher than many L-AAs and account for as much as one-third of L-serine levels” [376]. Free D-aspartic acid and D-alanine are found in the white and gray matter of healthy human brains [377]. d-Ser is known to be involved in glutamate transmission and plays a role in long-term potentiation [378]. D-AAs found in many AD-related proteins including neuronal-specific neurofilament-L [379], MBP [56], and in protein phosphatase PPC1 [380,381]. PPC1 and PPC2 are involved in TAU de-phosphorylation at multiple serine/threonine sites [382,383]. D-Ser was found to be involved in moto-neuron degeneration [384].

### 4.3. System Level: Morphological and Cognitive Aspects

#### 4.3.1. Aging, Long-Lived Proteins (LLP), and Racemization

“D-amino acids …. play a role in aging-related diseases associated with gradual protein racemization” [318].

Brain laterality is a complex phenomenon widely studied at molecular, cellular, brain morphological and functional levels. Age-related bio-chemical alterations of brain laterality are region, cell-type, and molecular biomarker (type/function) dependent and vary from increase, decrease, and reversal of hemispheric asymmetry [18,385]. Molecular and cellular determinants of an organism aging are evident from asymmetric cell division in embryo [135]. The proteins with a long lifetime have recently become the subject of increasing attention. The nucleoporins [386] and myelin-related proteins of oligodendrocytes [387,388] were identified as the most long-lived proteins in rodent brains. Age-related reduction in D-Ser level with age has been associated with deficiencies in cognitive ability [18,388–390]. The intracellular, membrane-bound, and extracellular proteins with long lifetime have been linked to the age-dependent cellular and organism levels of events including fertility and neurodegeneration [387]. Among known LLPs are α-synucleins [391], APP [392], and TAU [393], PrP [394], huntingtin [235], MBP [387] and collagen [87,381]. An accumulation of the altered forms of functional LLPs with aging [18,395] as well as age-dependent protein racemization are considered well-established facts [252]. Aging, at a molecular level is considered a collapse of homochirality of the entire organism including the eye (lens, ciliary body, drusen, and sclera), skin, cardiac muscle, blood vessels of the lung, and heart, stomach (chief cells, longitudinal and circular muscles), small and large intestines, and kidney [396,397]. In Fujii expression, molecular chirality is an “index of aging”. LLPs containing D-AAs are present at many sites in the human body including CNS. However, little is known about the major pathways of PTM that affect protein structure and function in the brain and the studies of link between the racemization, aging and aggregation of proteins are practically absent [194]. The modification and aggregation of functional proteins can be significantly influenced by age-related accumulation of abnormal enzymes [398]. In the prefrontal cortex of mammals (mouse, rat, human) at 1/3 gestation period, more than 50% of the aspartic acid is in D-configuration [399,400]. However, at the time of birth it becomes undetectable. It has been suggested that there is a role for D-Ser in the mechanism of neuronal death in the nervous system [113] that is also associated with pathological protein aggregation. Over the lifetime, a stochastic process leads to alteration of molecular chirality at the DNA and protein levels. The “molecular clock” of aging is influenced by the complex of genetic and epigenetic factors [401,402]. Particularly, a gradual racemization of peptide and protein has been observed in aging populations [318,403] both in humans and animals [379].

Both the proteins relevant to AD (such as TAU and Aβ), have been shown to contain many racemized AAs in brain tissue from elderly human donors [404,405].

The racemization of AAs affects TAU proteolysis and aggregation [317]. Age-related neuro-degradation processes are, to a significant degree, associated with the age-related protein degradation leading to accumulation of misfolding, dysfunctional aggregates. However, it is essential to understand that “protein aggregation is a normal physiological event” with an evolutionarily conserved mechanism balanced by the proteins degradation system [406,407]. It is obvious that distortion of the protein degradation systems will inevitably accelerate neurodegeneration. Autophagy is one of the cell type-specific degradation systems. Autophagy can be up/down-regulated upon many factors including starvation [408] and dietary exposing to environmental toxins [409].

Notably, such toxins can include the environmental/dietary D-AAs. The idea that AAs racemization can modulate proteolytic protein degradation is supported by many facts, some of which are indicated below.

- It is known that AAs composition is critical for aggregation-prone proteins [410] and PTM of AAs dramatically influences autophagic proteolysis [411,412].
- It was shown that the racemization results in the accumulation (aggregation) of altered proteins, accompanied by neurodegeneration [413,414].
- The serine-threonine kinase regulated autophagy and serine-proteases are functions in the signaling pathways [415–418].
- The specific serine proteases family (granzymes) which are expressed exclusively by cytotoxic T-lymphocytes and natural killer (NK) cells play a key role in apoptosis [419,420].

#### 4.3.2. Proteolysis vs. Aggregation: APP, Aβ and TAU

In the brain of AD patients, the Aβ peptide’s structural transition is initiated by the monomer to oligomer transition followed by conformation of the oligomers, protofibrils, fibrils, and plaques [3,421]. The detection of D-AAs in the A-β depositions and an affinity of A-β to D-peptides suggest the distinct role of L/D isomerism in the stages of the pathogenesis of neurodegeneration. The stages include a protein aggregation and plaque deposition [422,423]. D-AAs can be localized at the different positions in a peptide chain, including N- and C-termini. The D-AAs-containing peptides are resistant to proteolytic degradation suggesting the possibility of molecular aggregation and creating insoluble depositions [176,339,340]. The racemization of A-β and MBP was observed under different experimental conditions [101,424,425]. The increased level of DAAO was associated with the severity of the cognitive deficits in individuals with mild cognitive impairment and AD [83]. In brains of individuals with AD, D-Alanine (D-Ala) concentration is elevated more than twice [426]. D-Ser levels in the hippocampus and parietal cortex of AD patients are higher than in control subjects [427]. The comparison of physical and biological properties the all-D- and all-L-stereoisomers of Aβ (Aβ25–35) and the full-length peptide (Aβ1–42) reveal practically identical structural and assembly characteristics as well as similar levels of toxicity [428]. The deposition of abnormal protease-resistant proteins is presumably associated with the generation of D-AAs configuration [429]. The distribution of D- and L- aspartic and isoaspartic acids was studied in amyloid β peptides and TAU, designating new potential of the chiral biomarkers [430]. In 2006, Kokkoni et al. showed that ideal inhibitors of Aβ fibril are D-peptides [431] the conclusion is supported by later experiments [280,432]. The discovery of the effect of D-AAs peptides on beta-amyloid aggregation offers an attractive therapeutic strategy against protein misfolding diseases. Replacement of serine 422 with glutamic acid in TAU increases the propensity of TAU aggregation into insoluble fibril deposits of paired helical filaments (PHF) associated with neurodegeneration [433]. If we assume that D-AAs have function, then it is reasonable to link the decline of cognitive function with the changes in the balance between L- and D- AAs.

At present, the multiple isoforms of A-beta [434] and microtubule-associated proteins [435] are useful as biomarkers of neurodegenerative diseases. However, the potential usefulness of examining stereoisomers in protein synthesis and degradation pathways has far been under-appreciated. Recent developments reveal that the morphological and functional hemispheric lateralization and asymmetry originate from spontaneous intracellular symmetry breaking at the molecular and cellular level [436]. As a result, the primary physiological functions of the brain are asymmetrical between the left and right hemispheres. The morphological brain asymmetry correlates with cognitive functions [437,438]. A chain of lateralization is believed to originate from genetic as well as from epigenetic impact [21,439].

#### 4.3.3. Proteolysis

The degradation of proteins is modulated by many PTM pathways including AAs racemization. The specific SerPs family (granzymes) which are expressed exclusively by cytotoxic T-lymphocytes and natural killer (NK) cells play a crucial role in apoptosis [419,420]. It was shown that the racemization results in the accumulation of altered proteins, accompanied by neurodegeneration [413,414]. In AD, the general acceptance of the amyloid cascade hypothesis coexists with the failure of Aβ targeting drug therapy. Resolving this situation requires a broader view on the link between the variety of protein stereo-transformations and the multiplicity of degradation pathways. For example, the heterogeneity of cleavage sites of APP leads to a variety of Aβ peptides forms, of which only a small part of each (such as Aβ1–40 (Aβ40) and Aβ1–42 (Aβ42)) have been currently studied at the stereochemical level [440]. Only close attention to the interaction between the cleavage site of substrate and active site of enzyme will provide an insight to the molecular mechanism of enzyme activity and bring the key for predictive drug therapy [441–443]. One of the studies of pathways of APP proteolysis ^XXII^ involves the sequential cleavage by two aspartic proteases: β- and γ- secretases. (*XXII. Currently about 570 human proteases listed in the human Degradome Database*).

However, it has become obvious that the β-/γ- secretases pathway of protein degradation represents only the “tip of the iceberg” complemented by many alternatives. Among them are the proteinase families of hydrolytic enzymes including SerPs, glutamic acid proteases, and metallo-proteinases. All SerPs enzymes (including trypsin, chymotrypsin, elastate, thrombin, subtilisin, plasmin, TPA, and factor D) contain a “catalytic triad” of Ser, His, and Asp. From this perspective, SerPs represent an attractive subject of exploration due to the combination of two facts: (1) their role in the lysosomal-endosomal protein degradation, and (2) expected effect of the racemization. However, SerPs role in the APP and TAU processing is not clearly understood. The SerPs (cathepsin A and G), aspartic proteases (cathepsin D and E) and cysteine proteases (cathepsins (B, C, L, F, H, K, O, S, V, X, and W) belong to proteinase families of hydrolytic enzymes. The proteinase families of hydrolytic enzymes are classified based on the mechanism of catalytic activity as aspartic, metallo, cysteine, serine, or threonine proteases [444,445]. The cathepsins are expressed in the brain in a cell type-specific manner. The activity of serine-cysteine protease was detected within the phagosomes of macrophages [446].

The enzyme activity is strongly influenced by the racemization of active AAs residues such as aspartic acid, threonine, and serine, causing AAs cross-linking and aggregation. It is believed that the deposition of abnormal protease-resistant proteins is associated with the generation of D-AAs [329].

#### 4.3.4. Revision of Aggregation Hypothesis

“Protein aggregation may be exploited by nature to perform specific physiological functions” [406].

“Replacement of serine 422 with glutamic acid in TAU increases the propensity of tau aggregation associated with neurodegeneration” [272].

“TAU Phosphorylation at Ser 422 is observed from the earliest stages of TAU aggregation” [447].

Close attention of researchers to the link between protein aggregation and PTM associated with the Ser residues is evident in the current flow of publications [262,406,447]. We mentioned before that the various PTMs of proteins and peptides (including Aβ) were explored regarding age-related degradation processes [297]. It is not surprising that all hypotheses of protein aggregation in AD are directly or indirectly associated with D-AAs metabolism. Among them are the following: amyloid [448], cholinergic [449,450], proteases [451], N-terminal [452,453], oxidative stress [454,455], branched-chain AAs [456], and amyloid-β crosslinking [457] hypotheses. In these circumstances, the racemization hypothesis of protein aggregation naturally gains its legitimacy. Recent results show that the mutation in D-amino acid oxidase (D-AAO) gene associated with familial ALS impairs D-Ser metabolism and causes protein aggregation [120], suggesting a close association between protein folding and D-AAs metabolism.

It is notable that D-Ser is predominantly released from glia cells (protoplasmic type II astrocytes). These cells enclose nerve terminals and are enriched in specific regions of the gray matter including cerebral cortex, hippocampus, anterior olfactory nucleus, olfactory tubercle, and amygdala [98,228,458,459]. In the brain of AD individuals, the chain of Aβ peptide structural transitions is initiated by the monomer to oligomer transition followed by the protofibrils, fibrils, and plaques formation [3,421]. The studies of the early stage of Aβ42 monomer aggregation reveals the co-existence of two distinct pools of stereo conformation: locally structured “A” and disordered “B” states [460]. The detection of D-AAs in the A-β depositions and an affinity of A-β to D-peptides suggest the distinct role of L/D isomerism in the stages of neurodegeneration. These stages include protein aggregation and plaque deposition [422,423]. D-AAs can be localized at the different position in a peptide chain, including N- and C-termini. The D-AAs-containing peptides are resistant to proteolytic degradation suggesting the possibility of molecular aggregation and insoluble depositions [321,339,340].

The increased level of DAAO was associated with the severity of the cognitive deficits in individuals with mild cognitive impairment and AD [83]. In brains of AD individuals with D-Alanine (D-Ala), the concentration is elevated more than twice [426]. D-Ser levels in the hippocampus and parietal cortex of AD patients are higher than those in control subjects [427]. Comparison of the physical and biological properties of all-D- and all-L- stereoisomers of Aβ (Aβ25–35) and the full-length peptide (Aβ1–42) reveals practically identical structural and assembly characteristics as well as similar levels of toxicity [428].

## 5. Treatment of Protein Aggregates

The discovery of the effect of D-AAs on Aβ aggregation offers an attractive therapeutic strategy against protein misfolding diseases. Notably, Ser is one of three AA residues (in addition to threonine, and tyrosine) commonly phosphorylated during cell signaling in eukaryotes. Phospho-serine is a component of many proteins as the result of PTM by various types of kinases (more than 50) [461,462]. Hyper phosphorylated TAU is the second major feature of AD. According to contemporary view the network of PTM of TAU protein (monomer) is causal for the assembly of monomers into diverse forms [159,463]. The major forms of aggregation are: oligomers, paired helical filaments (PHFs) and neurofibrillary tangles (NFTs). At a structural level, NFTs consist of PHFs. Among the different TAU isoforms are the neuron-protective and neuron-toxic subsets [464–466]. Side-specific phosphorylation of TAU can lead to formation of functional and neuro-protective (inhibits amyloid-β toxicity) iso-forms [465]. Neurodegenerative TAU-pathy is characterized by the hyper-phosphorylation of all TAU isoforms. The PHFs and NFTs do not play a role as the toxic entities leading to disease. The toxicity is ascribed primarily to the TAU oligomer [464]. Hyper-phosphorylated forms of TAU were identified in neuronal somata, neuropil threads, and plaque-like clusters of neuritis [467]. Ser and threonine residues are among the primary targets of phosphorylation. A serine/threonine-proline kinase phosphorylates TAU proteins stereo-transformation forming a paired helical filament [468]. The phosphorylation of TAU is required for hippocampal LTD [469]. TAU protein contains serine 202, 395, and 404 and threonine 205 and 394 residues as targets of differential PTMs [469–471]. The replacement of AAs in TAU increases the propensity of TAU aggregation [433].

In summary, the above-mentioned factors play a role in the evolution-supported association between biochemical events, behavioral patterns, and cognitive functions. The aggregation of the β-amyloid (Aβ) peptide into toxic oligomers is a key pathogenic event in the AD. The fact that dietary exposure to the L-Ser containing products reduces the risk of NFT and β-amyloid deposits in the brain suggests the essential role of AAs racemization on neurodegenerative diseases (NDs) [409]. The current strategy for prevention and treatment of existing protein aggregates and their toxicity is aimed at the stereoselective properties of the D-enantiomeric acids and peptides for TAU and Aβ [423,472] associated depositions.

The essential finding is that Aβ42 exhibits an affinity to the D-AAs peptides [472]. The molecules that interfere with aggregation and toxicity potentially may act as therapeutic agents for the treatment of the disease. Many D-AA peptides exhibit an ability to inhibit or promote protein aggregation depending on the binding site [473–475]. The studies of the molecular inhibitors of A-beta aggregation successfully used the short peptide fragments homologous to the specific fragment-sequence of full-length wild-type A-beta. It was shown that the effectiveness of the inhibitors is strongly attenuated by replacement of L- to D-AAs or methylation of Aβ fragments [65,431,476].

## 6. Racemization Hypothesis

Biological evolution has predominantly selected one structural form for AAs (L- levorotary form not the D- dextrorotary form). The consequence of this selection is that proteins being formed will primarily consist of L-AAs. Correspondingly, the enzymes involved in PTM and metabolism of proteins will primarily handle and metabolize (although not completely exclusively) the L- form.

Contemporary studies have revealed that the presence of D-AAs in the organism is not accidental and has fundamental importance for the function of the CNS and adaptation of the single cell and entire organism to the stereochemical environment. The genetic and epigenetic disturbance of the natural balance in the concentration of the free and protein/peptide bound L- and D- AAs in the brain (and or in peptide composition) leads to the misfolding of the D-AAs-containing proteins. This is incompatible with the evolutionary design of protein synthesis, degradation, and repair mechanisms including autophagy [478]. The interference of the spontaneous, enzyme-driven, and environmentally induced racemization can disrupt the functional structure of proteins leading to adverse effects on biological activity [244]. The exploration of the AAs racemization [28,266] and a protein aggregation within the bacterial cells opens an evolutionary perspective on human pathology. The combination of new results suggests specific attention to AAs racemization. It is obvious that along with the functional proteins, most of the enzymes, including Ser-proteases [202] and gamma-secretase [50,479] are involved in the process of AA racemization and APP proteolysis in proteins containing Ser and Asp residues. The fact that both residues are the subject of age dependent racemization allows one to assume an aging effect in enzyme activity. Indeed, it has been found that enzymes undergo age-related modifications which include structural changes and their specific affinity [480–482]. The prevalence of proteins receiving non-enzymatic PTM was found to be “increased with aging and is thought to be closely related to age-associated changes” [89]. Since 1975, the racemization of AAs in proteins has been used as a means of assessing the “age” of proteins [89,483]. However, in the current research, the aging of enzymes is not considered usually in respect to the process of racemization and protein aggregation.

## 7. Conclusions

The internal molecular environment of living systems is characterized by the specific structure–function relationships evident in the activity of signaling proteins, transporters, enzymes, DNA, and RNA. The discovery of a “shape-shifting” molecule (SSM) that is capable of interconversion among thousands of structural isomers has ascertained the dynamic nature of molecular chirality [14]. The stereoselective metabolism of chiral biomolecules emphasizes the significance of the effect of racemization in protein misfolding and aggregation.

We want in this overview to draw attention to the need to further examine the following points:

What is the mechanism linking the residue-specific protein racemization with aggregation?

How does racemization at specific sites contribute to protein aggregation, deposition, and toxicity associated with the major neurodegenerative disorders?

What are the functional consequences of site-specific racemization of A-beta, TAU, Huntingtin, α-synuclein, PrP, and MBP?

Do the residue-specific modulators (inhibitors and enhancers) of racemization have beneficial therapeutic effects?

How does the interplay between the enzymatic and spontaneous PTMs influence protein aggregation in neurodegenerative diseases [484].

For several decades, most treatments for AD have been targeted against the amyloid-β (Aβ) peptide. The frequently asked question is “why this strategy fails” [485–487]. The answer lies in the neglect of the link between AAs chirality, the stereochemistry of protein folding, neurodegeneration, and cognitive decline.

We are confident that the review of available information provides the conceptual and experimental background to understand the phenomena of protein aggregation indicating that racemization significantly contributes to brain pathology, and its integrated study would reveal novel therapeutic procedures. The role of racemization in decline of cognitive functions should be studied in conjunction with the major cellular players of protein aggregation pathology. Among them, prior consideration should be given to the trans-membrane receptors (NMDA, AMPA, mGlu5, α7, nAChRs, NGF, and apoE receptors (including receptors in neurons of the olfactory epithelium) [116]), glycoproteins [48], and cell membrane constituents (including cholesterol, and collagen).

The chiral cholesterol was shown to be a mediator of the stereoselective interaction between the cell membrane and proteins [488]. Therefore, the cholesterol of synaptic spine underlines the mechanism of glutamatergic neurotransmission. At the extracellular domain, the molecular mechanisms involved in the collagen aging and aggregation (cross-linking) are also significantly contributed by racemization [87,381]. The dynamic protein chirality, in our view, is a significant determinant of lateral asymmetry of neurotransmitters in the human brain [488].

## Abbreviations

AAs: Amino Acids
Aβ: Amyloid beta
APP: Amyloid precursor protein
BACE1: β-secretase 1
BM: Biomarker
D-Ala: D-Alanine
D-AAs: D-amino acids
D-Ser: D-serine
EM: Electron microscopy
ND: Neurodegenerative diseases
TEM: Transmission electron microscopy
SEM: Scanning electron microscopy
VCD: Vibrational circular dichroism
MPs: Misfolded proteins
PHFs: Paired helical filaments
PTMs: Post-translational modifications
PTM-Sys: System of post translational modification
SerR: Serine racemase
SSM: Shape-shifting molecule
